# 

**Published:** 1878-12-20

**Authors:** 


ORIGINAL AND SELECTED ARTICLES.

PREPARATION OF PEPSIN AND THE GOOD TO BE OBTAINED FROM IT: by Joseph Adolphus, M. D., of Ga. .......... 369
Turning in Arm Presentations; by A. G. Hobbs, M. D., of I d, .......... 372
Diarrhœas of Children .......... 372
Microscopical observations in Yellow fever; by J. B. Marvin, M. D., of Ky .......... 373
Congenital Malformation; by L. G. Hardman, M. D., of Ga .......... 375
Neuralgia and other disorders caused by uterine disease; by D. N. Kinsman, M. D., of Ohio .......... 376
The treatment of Ulcers with a saturated solution of the Chlorate of Potassium; by T. M. Rochester, M. D., N. Y .......... 380
Compound Comminuted fracture into the ankle joint; irrigation with alcoholic solution of coal tar; rapid recovery .......... 382

ABSTRACTS AND GLEANINGS.

Dermalgia, etc., from quinine, 383. Comparison of Opium, Belladonna and Aconite, 384. Iodoform for Syphilitic Sores, 385. Treatment of Tinea Favosa, 385. On the use of Calcium Chloride in the treatment of Phthisis, tabes mesenterica, chronic Diarrhœa, etc. 386. Tubercular ulcer of the Tongue, 386. Tartarized Antimony, 397. Treatment of Nævus by Sodium Ethylate, 387. Spinal Curvatures, 388. Ointment of Nitrate of Silver, 383. Intestinal gas-flatulent Dyspepsia, 389. Ipecac, 389. Ovariotomy Superseded, 389. Urea formation, 389. Treatment of Diarrhœa by oxide of Zinc, 390. Fibroid tumors of the Uterus by Sclerotic acid, 390. Death from Chloroform, 390. Treatment of Ptyriasis Versicolar, 391. Acetic injections in Carcinoma, 391. Treatment of intertrigo in Children, 391. Sulphurous Acid for Abscesses, 391. Treatment of Sore Nipples, 392. Pyrogallic Acid in Psoriasis, 392. Liquor San'al Flava cum buchu et cubeba, 392. Codeia for Cancer of the Stomach, 392. Nitrate of Amyl, Chloroform Narcosis. Lead washes for the Eye, 392. Pancreatic fluid; Ovarian Dyspepsia; Iodoform; Bi. Carb. Soda; Vulvar Pruritus, 393.

PRACTICAL NOTES AND FORMULÆ.

Note from G. L. Glazener, M. D, of S. C., on balsam of fir in wounds; treatment of Dyspepsia, with formulæ, &c., 394. Chloral hydrate locally applied; Impotence and Spermatorrhœa; Chlorymal; Paint for rough wood work; Iodoformized cod liver oil, 395; Urticaria; Prescription for Amenorrhœa; Anti-rheumatic Pills, 396. Bitters; to keep flies from sores on domestic animals; Ointment for tetter; frosted feet and hands; catarrh snuff; pimple cerate, 396.

SCIENTIFIC ITEMS.

Draper's Scientific Memoirs; Edison; Evolution, 397.

EDITORIAL AND MISCELLANEOUS.

Emulation, 398. A New Medical College in Atlanta, 399. Our Journal for 1879; Pharmaceutical Association; Dr. Luke P. Blackburn; Receipts, 400.

